# *MGMT* Promoter and Enhancer Methylation in Melanoma Brain Metastases and Glioblastoma: Shared and Distinct Features

**DOI:** 10.3390/cells15050410

**Published:** 2026-02-26

**Authors:** Katharina Pühringer, Benno Fehringer, Katja Zappe, Walter Berger, Serge Weis, Sabine Spiegl-Kreinecker, Margit Cichna-Markl

**Affiliations:** 1 Institute of Analytical Chemistry, Faculty of Chemistry, University of Vienna, 1090 Vienna, Austria; 2 Vienna Doctoral School in Chemistry (DoSChem), University of Vienna, 1090 Vienna, Austria; 3 Center for Cancer Research and Comprehensive Cancer Center, Medical University of Vienna, 1090 Vienna, Austria; 4 Division of Neuropathology, Department of Pathology and Molecular Pathology, Kepler University Hospital GmbH, Johannes Kepler University, 4040 Linz, Austria; 5 Clinical Research Institute for Neurosciences, Johannes Kepler University, 4020 Linz, Austria; 6 Department of Neurosurgery, Kepler University Hospital GmbH, Johannes Kepler University, 4040 Linz, Austria

**Keywords:** Melanoma brain metastases, glioblastoma, DNA methylation, MGMT, promoter, enhancer, pyrosequencing

## Abstract

Many cancer-associated deaths result from metastases rather than primary tumors. Growing evidence suggests that DNA methylation alterations are crucial for inducing a plastic phenotype that allows cancer cells to adapt to the metastatic microenvironment. Brain metastases of melanoma (MBM) and glioblastoma (GB) share a neuroectodermal origin and the brain as tissue of residence, but their epigenetic regulation is poorly understood. Aiming at elucidating shared and tumor-distinct features, we analyzed the methylation of *MGMT* regulatory elements. We focused on *MGMT* because *MGMT* promoter methylation is used as a predictive marker for temozolomide response in GB, but its role in MBM has been discussed controversially. By targeting 12 CpG dinucleotides (CpGs) in the promoter, 68 CpGs in intergenic enhancers, and 31 CpGs in intragenic enhancers, we identified shared features, including an L-shaped relationship between promoter methylation and MGMT protein expression and an inverse L-shaped relationship between intragenic enhancer methylation and MGMT protein expression. GB exhibited higher methylation, particularly in promoter and intergenic enhancers, and stronger associations between methylation and overall survival than MBM. These results highlight both conserved and tumor-specific *MGMT* regulation, reflecting the complexity of epigenetic control in brain malignancies and emphasizing divergent evolution between MBM and GB.

## 1. Introduction

Many cancer patients die from metastases rather than from their primary tumors [1]. Metastasis is a multi-step process that requires tumor cells to disseminate, seed, and colonize distant organs [2]. To succeed, cells must adopt a plastic phenotype that allows them to adapt to the microenvironment of the metastatic site, interact with resident cells, and evade immune surveillance [1,3]. Increasing evidence suggests that these traits are shaped not only by genetic but also by epigenetic mechanisms [4,5].

Brain metastases (BM) most commonly arise from lung, breast, kidney, or melanoma and are the most frequent and among the deadliest brain tumors [6]. Despite therapeutic advances, median overall survival (OS) remains poor [7]. BM are particularly challenging due to limited drug penetration across the blood-brain barrier and distinct mutations compared to the primary tumor [4,7,8].

Genome-wide methylation studies have revealed differences in the methylome between metastases and primary tumors [8,9], across metastatic origins [10], and between intracranial and extracranial metastases from the same origin [11]. In addition, distinct methylation signatures have been reported for BM versus glioma [9].

In this study, we aimed at exploring shared and distinct DNA methylation patterns between brain metastases of melanoma (MBM) and glioblastoma (GB). Both tumors arise from lineages of neuroectodermal origin: melanocytes and glial cells are derived from neural crest cells, which originate from the neuroectodermal layer [12]. The common origin might contribute to the strong propensity to metastasize to the brain [13]. In a targeted approach, we focused on methylation of regulatory elements of *MGMT,* which encodes O6–methylguanine methyltransferase, a DNA repair protein that removes the cytotoxic lesion O6–methylguanine [14,15]. Tumors with elevated MGMT expression frequently show decreased sensitivity to DNA-alkylating agents, including temozolomide (TMZ) [16]. In GB, promoter methylation of *MGMT* predicts therapeutic response to TMZ, particularly in recently diagnosed older patients [17].

In contrast, MBM patients rarely respond to TMZ [18,19,20,21], and the relevance of *MGMT* promoter methylation as a predictive biomarker remains controversial [19,22]. In addition to methylation of regulatory elements of *MGMT*, we were interested in methylation of Long Interspersed Nuclear Element-1 (LINE-1) as a surrogate marker for global DNA methylation. By elucidating shared and distinct features of *MGMT* promoter and enhancer methylation and global DNA methylation in MBM and GB, we aimed to advance our current knowledge of *MGMT* regulation in these two different brain malignancies. In addition, *MGMT* enhancer methylation was analyzed in a small set of primary melanoma samples for exploratory comparison with MBM.

## 2. Materials and Methods

### 2.1. Samples and Cell Culturing

Approval for this study was granted by the Ethics Commission of the Faculty of Medicine at Johannes Kepler University Linz (application number E-39-15). All patients signed written informed consent forms prior to participation. Melanoma brain metastases samples originate from the Department of Neurosurgery, Kepler University Hospital, Linz, where patients underwent surgery between 2001 and 2020. All analyses were performed on immortalized cell cultures established from surgically removed metastases. Primary melanoma cell cultures were obtained from surgical samples from lesions with different histologies as published [23] and used at low passage numbers. Cells were maintained in RPMI-1640 supplemented with 7% fetal calf serum (FCS) and 1% glutamine, without antibiotics (Sigma-Aldrich, Darmstadt, Germany), in a humidified incubator with 5% CO_2_ at 37 °C (Binder, Tuttlingen, Germany). Cells were harvested before reaching confluence between passages 5 and 10, pelleted by centrifugation, and stored at −80 °C until DNA extraction.

### 2.2. Determination of Genetic Variants and Clinical Parameters

MGMT protein levels were assessed by Western blot and quantified relative to β-Actin. Expression levels of mRNA were assessed through semiquantitative real-time PCR using GAPDH as a housekeeping gene.

Mutations in the *TERT* promoter (C228T, C250T, C242T, and C243T) as well as *TERT* SNP rs2853669 genotypes (T < C < G) were identified by sequencing using BigDye Terminator v1.1 Cycle Sequencing Kit (Applied Biosystems, Waltham, MA, USA) and a 3130 Genetic Analyzer (Applied Biosystems, Waltham, MA, USA) following standard procedures [24]. Information on the age and sex of the patients was available.

### 2.3. DNA Methylation Analysis

**Figure 1:**
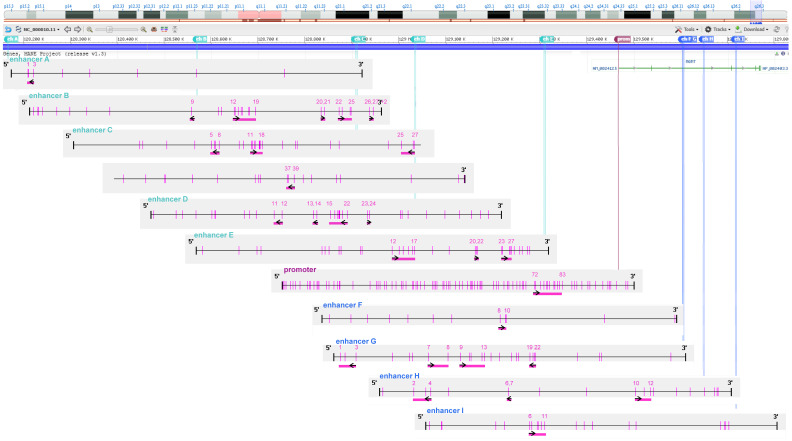
Overview of targeted CpG sites in *MGMT* regulatory regions. Intergenic enhancers are shown in turquoise, intragenic enhancers in blue, and the *MGMT* promoter in dark pink. Vertical pink lines indicate CpG dinucleotides. Horizontal pink bars mark sites that were analyzed by pyrosequencing. The sequencing direction relative to the upper strand is indicated by black arrows. CpGs were numbered according to their position in the promoter/enhancer sequence. CpG positions within the regulatory elements were visualized using Methyl Primer Express Software v1.0 (Thermo Scientific, Vienna, Austria). The location of *MGMT* on chromosome 10 was retrieved from the NCBI Genome Data Viewer [29]. Intergenic enhancers: Enhancer A (hs542): 1032 bp, 8 CpGs; enhancer B (hs737): 1138 bp, 27 CpGs; enhancer C (Chen et al. [28]: 3313 bp, 46 CpGs, CpGs 1–25: “Del 1”, CpGs 26–46: “Del2”; enhancer D (hs699): 1719 bp, 33 CpGs; enhancer E (hs562): 2221 bp, 32 CpGs. Intragenic enhancers: Enhancer F (hs656): 1332 bp, 12 CpGs; enhancer G (hs696): 1243 bp, 26 CpGs; enhancer H (hs331): 1899 bp, 20 CpGs; enhancer I (hs589): 1366 bp, 21 CpGs. prom: promoter, eh: enhancer.

DNA methylation analysis of CpGs in the *MGMT* promoter and nine enhancers of *MGMT* was carried out as described previously [25,26]. The enhancers are referred to as enhancer A–I, according to their genomic position, including intergenic enhancers A (hs542 [27]), B (hs737 [27]), C (identified by Chen et al. [28], D (hs699 [27]), and E (hs562 [27]) and intragenic enhancers F (hs656 [27]), G (hs696 [27], H (hs331 [27]), and I (hs589 [27]). Enhancers F, G, and H are located in intron 2; enhancer I is located in intron 3 of the *MGMT* gene. Additionally, we investigated the methylation status of five CpGs within the LINE-1 element (human LINE-1 transposon), which serves as a marker for global DNA methylation. The LINE-1 assay was developed in this study based on NCBI accession number X58075.1. A summary of all analyzed regulatory elements, respective coordinates, size, and number of CpGs is given in Supplementary Table S1. Primer sequences of forward, reverse, and sequencing primers are given in Supplementary Table S2. Figure 1 gives a graphical overview of all regions analyzed in the present study.

In brief, DNA extraction from pelleted cells was performed using the QIAamp DNA Blood Mini Kit (Qiagen, Hilden, Germany). Next, DNA was treated with bisulfite to convert unmethylated cytosines into uracil, followed by a cleanup of the products (EpiTect Fast Bisulfite Conversion Kit (Qiagen, Hilden, Germany)). DNA was quantified using the Qubit 4 fluorometer, following the manufacturer’s protocol with the Qubit ssDNA Assay Kit (Thermo Scientific, Vienna, Austria). Target regions for the promoter and enhancers were amplified by real-time polymerase chain reaction (PCR), using 5 ng of DNA. Methylation levels at CpG sites were assessed by pyrosequencing (PyroMark Q48 autoprep system, PyroMark Q48 Accessories, and PyroMarkQ48 Advanced CpG Reagents (Qiagen, Hilden, Germany)).

### 2.4. Data Analysis and Statistics

Data analysis and creation of graphics were performed using R software (R Foundation for Statistical Computing, Vienna, Austria; version 4.3.1). Differences between two groups were assessed using the Mann-Whitney U test. For assessing differences among more than two groups, the Kruskal-Wallis H test followed by post-hoc pairwise Mann-Whitney U tests was applied. Spearman correlation coefficients were calculated to assess correlations between variables. Standardized effect sizes for comparisons of CpG methylation levels between primary melanoma and melanoma brain metastasis cohorts were calculated as Cohen’s d and corrected for small-sample bias using Hedges’ correction (Hedges’ g). Analyses were hypothesis-driven and focused on predefined CpG sites within the *MGMT* regulatory regions. Therefore, no correction for multiple testing was applied, unless stated otherwise. *p*-values ≤ 0.05 were considered statistically significant.

Cut-offs for categorizing CpGs as methylated or unmethylated were defined individually for each site based on the observed methylation distribution in the sample set. Thresholds were adjusted to account for the specific methylation range of each CpG while ensuring that the resulting methylated and unmethylated groups were of roughly comparable size, avoiding groups with only one or two samples.

## 3. Results

### 3.1. Methylation Levels of MGMT Promoter, Intergenic and Intragenic Enhancers

**Figure 2:**
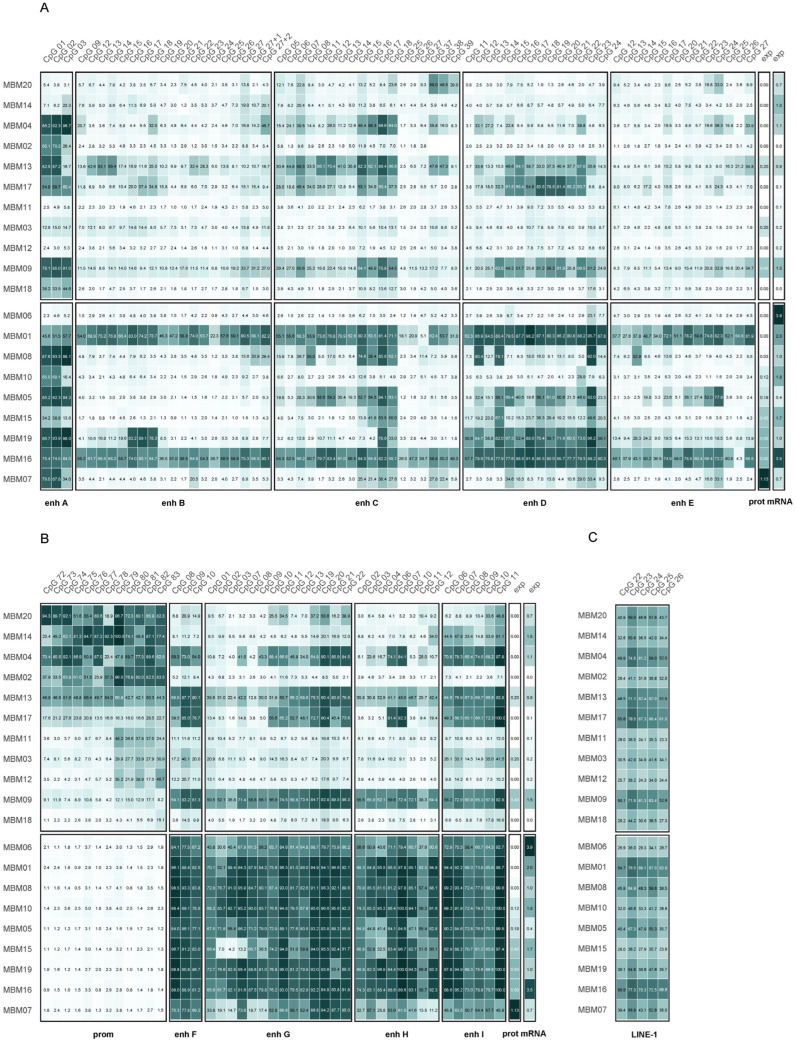
Heatmap of patient-specific methylation levels in MBM. Methylation levels of the *MGMT* promoter, intergenic and intragenic enhancers, and LINE-1 are shown. (**A**) Intergenic enhancers A–E. (**B**) *MGMT* promoter and intragenic enhancers F–I. (**C**) LINE-1. Samples are grouped by *MGMT* promoter methylation status (upper panel: methylated; lower panel: unmethylated) and further sorted by MGMT protein expression levels. Values represent means of two independent PCR/PSQ experiments. Color scale: white to dark green (0–100% methylation). prot: protein, prom: promoter, enh: enhancer.

DNA methylation was analyzed by pyrosequencing, covering 12 CpGs (CpGs 72–83) in the *MGMT* promoter and 99 CpGs in enhancer regions. Sixty-eight of the enhancer CpGs are located upstream of the *MGMT* gene and 31 CpGs within the gene (Figure 2). DNA methylation analysis of 20 MBM samples (Table 1) was done in this study; methylation levels in GB samples were determined previously [25,26]. Characteristics of the GB patient cohort are summarized in Table S3. All GB samples included in this study were IDH-wildtype. Distributions of age, sex, and OS of patients of the two cohorts are shown in Figure S1.

In nine (45.0%) MBM samples, the *MGMT* promoter was unmethylated (methylation < 5%), and in eleven (55.0%) samples, it was methylated (Figure 2). In promoter methylated samples, the mean methylation of individual CpGs ranged from 26.4% (CpG 78) to 49.7% (CpG 79), median methylation ranged from 13.5% (CpG 77) to 47.8% (CpG 79).

**Figure 3:**
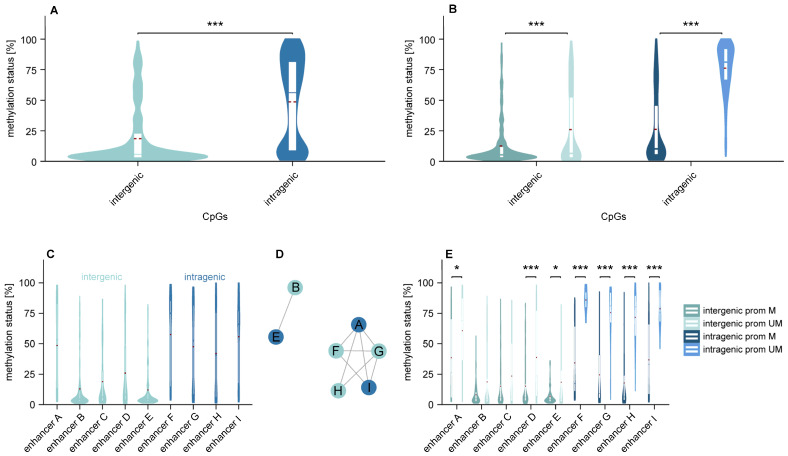
Distinct methylation patterns across intergenic and intragenic enhancers in MBM. (**A**–**C**,**E**) Violin plots showing CpG methylation distributions in intergenic versus intragenic enhancers. (**A**,**B**) Methylation distribution of the 68 intergenic enhancer CpGs versus the 31 intragenic enhancer CpGs. Samples are shown without stratification (**A**) or stratified by *MGMT* promoter methylation status (**B**). (**C**,**E**) Methylation distribution of the CpGs in the individual enhancers, without sample stratification (**C**) or stratified by *MGMT* promoter methylation status (**E**). (**D**) Network graph illustrating correlations among enhancer methylation levels, highlighting similarity between enhancers B and E, and among enhancers A, F, G, H, and I. Mean: red dashed line, * *p* ≤ 0.05, *** *p* < 0.001. Abbreviations: prom UM: *MGMT* promoter-unmethylated, prom M: *MGMT* promoter methylated.

Intragenic CpGs showed higher methylation than intergenic CpGs (*p* < 0.001; Figure 3A). Intergenic CpGs followed a unimodal distribution, whereas intragenic CpGs displayed a bimodal distribution (Figure 3A). Both intergenic and intragenic enhancers were more methylated in *MGMT* promoter-unmethylated than in promoter-methylated samples (*p* < 0.001; Figure 3B). We observed distinct co-methylation patterns of enhancers, with enhancers B and E clustering together and enhancers A, F, G, H, and I forming a second group (Figure 3C,D). Significant differences between promoter-methylated and promoter-unmethylated MBM were found in intergenic enhancers A (*p* ≤ 0.05), D (*p* < 0.001), and E (*p* ≤ 0.05), and intragenic enhancers F–I (*p* < 0.001; Figure 3E).

**Figure 4:**
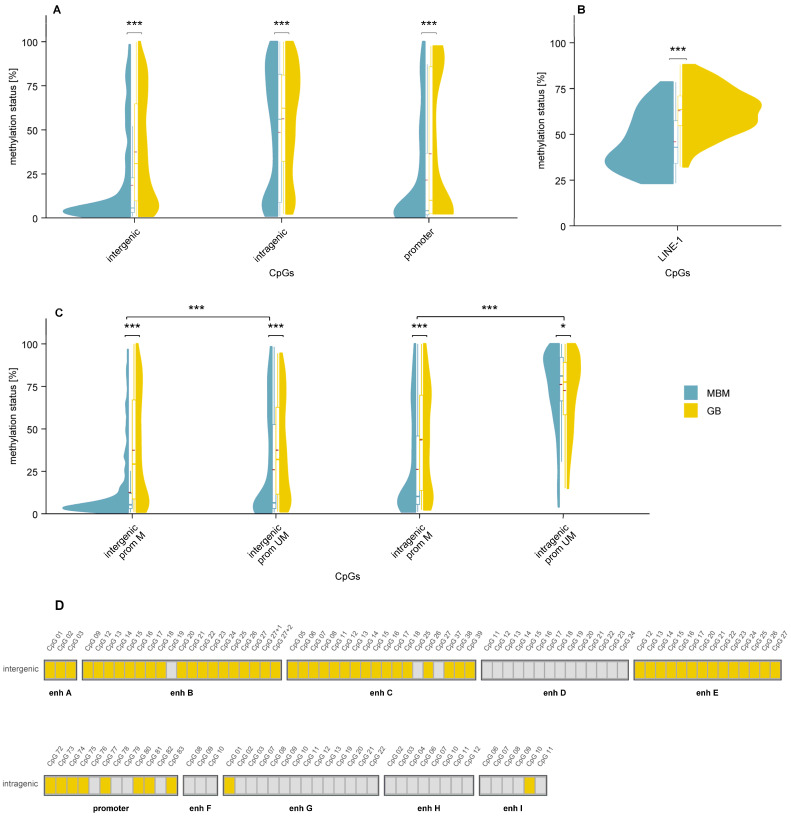
Differential methylation in MBM and GB samples. (**A**,**C**) Violin plots showing CpG methylation distributions in intergenic and intragenic enhancers and in the *MGMT* promoter. (**A**) All samples, (**C**) samples stratified by *MGMT* promoter methylation status. (**B**) Violin plot of LINE-1 methylation. MBM samples are shown in turquoise, GB samples in yellow. For all three regulatory element groups and LINE-1, MBM displayed consistently lower methylation than GB. Mean: red dashed line, * *p* ≤ 0.05, *** *p* < 0.001. (**D**) CpG-wise comparison between MBM and GB, with each box representing one CpG. Yellow indicates significantly higher methylation in GB, and grey indicates no significant difference between MBM and GB. Abbreviations: prom UM: *MGMT* promoter-unmethylated, prom M: *MGMT* promoter methylated, enh: enhancer.

Comparison of MBM and GB data revealed consistently lower methylation in MBM than GB samples across all regulatory elements analyzed (Figure 4A). Differences in enhancer methylation remained significant when MBM and GB samples were stratified by *MGMT* promoter methylation status (Figure 4C). Differences between MBM and GB were most pronounced in the *MGMT* promoter and intergenic enhancers A–C and E, with 59 (73.8%) of 80 CpGs being significantly lower methylated in MBM (*p* ≤ 0.05; Figure 4D).

In addition, DNA methylation in six independent primary melanoma samples was analyzed for comparison with MBM. Clinical characteristics and detailed methylation data are provided in Supplementary Table S4 and Supplementary Figures S2 and S3. Overall, CpGs located in both intergenic and intragenic *MGMT* enhancers showed lower methylation levels in MBM compared to primary melanoma. In contrast, higher methylation levels in MBM were observed for CpG sites in intergenic enhancer D and the *MGMT* promoter (Supplementary Figure S3, Tables S5 and S6).

### 3.2. Global DNA Methylation

Global DNA methylation was assessed by targeting five CpGs in LINE-1 by pyrosequencing in MBM (Figure 2C) and GB (Figure S4). LINE-1 methylation was significantly lower in MBM than in GB samples (*p* < 0.001, Figure 4B), indicating lower global DNA methylation in MBM. By contrast, LINE-1 methylation levels in MBM were higher than those observed in the independent primary melanoma cohort (Supplementary Table S5, Supplementary Figure S3).

### 3.3. Association of Enhancer Methylation with CpG Position

**Figure 5:**
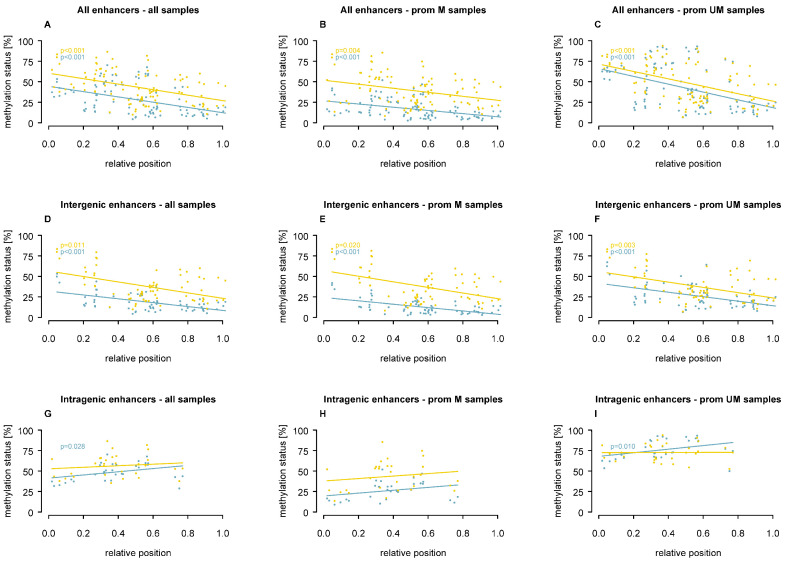
Association of enhancer methylation with relative CpG position in MBM and GB. Plots show methylation levels in relation to CpG position for MBM (turquoise) and GB (yellow). (**A**–**C**) all enhancers, (**D**–**F**) intergenic enhancers, (**G**–**I**) intragenic enhancers. Without sample stratification (**A**,**D**,**G**), *MGMT* promoter-methylated samples (**B**,**E**,**H**), and *MGMT* promoter-unmethylated samples (**C**,**F**,**I**).

Compared to the *MGMT* promoter, CpG density in enhancers was much lower, with the CpG content ranging from 1.6% (enhancer A) to 4.8% (enhancer B). Analysis of CpG position within enhancers (Figure 5) revealed that, in both MBM and GB, CpGs closer to the 5′ end of intergenic enhancers were more highly methylated compared to those at the 3′ end (*p* ≤ 0.020, Figure 5D–F), regardless of promoter methylation status. In contrast, intragenic enhancers showed an inverse association in MBM, where CpGs at the 3’ end were more highly methylated, when all samples (*p* = 0.028, Figure 5G) or promoter-unmethylated samples only (*p* = 0.010, Figure 5I) were included. These findings suggest that the relative position of CpGs may have an impact on distinct enhancer methylation patterns.

### 3.4. Association Between MGMT Promoter and Enhancer Methylation

**Figure 6:**
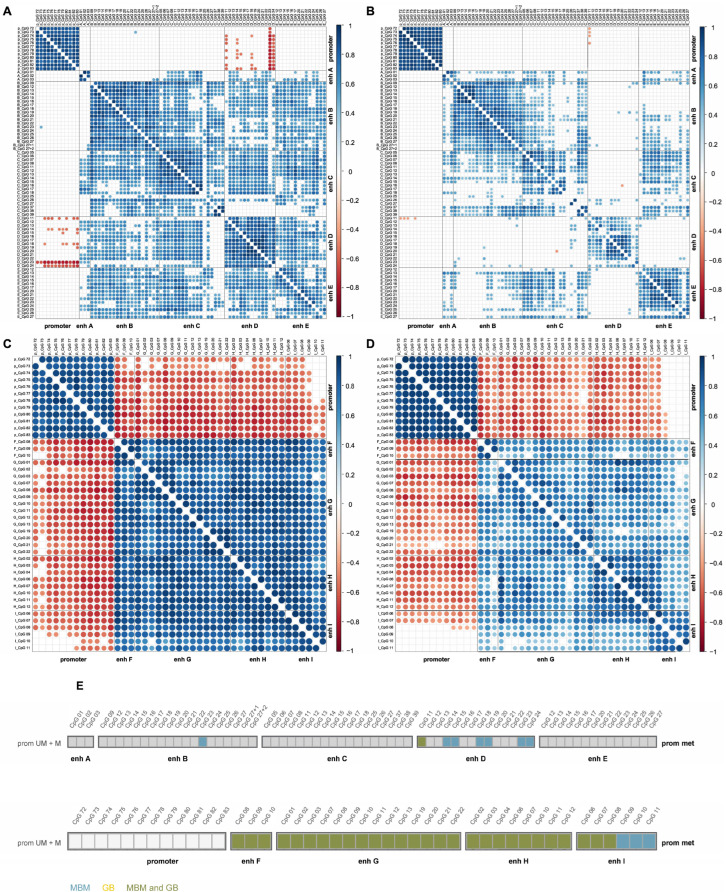
(**A**–**D**) Correlations between *MGMT* promoter and enhancer methylation in MBM and GB. Spearman correlation matrices for intergenic enhancers (**A**,**B**) and intragenic enhancers (**C**,**D**) in MBM (**A**,**C**) and GB (**B**,**D**). Significant positive and negative correlations are indicated by color intensity (dark red, −1.0; dark blue, 1.0; Benjamini–Hochberg adjusted *p* values). (**E**) Summary of significant correlations between methylation of enhancer CpG sites and *MGMT* promoter CpGs, with each box representing one CpG. Turquoise: MBM-specific, yellow: GB-specific, green: shared between MBM and GB. Abbreviations: prom UM + M: *MGMT* promoter-unmethylated and methylated samples, enh: enhancer.

We next analyzed correlations of CpG methylation levels within and across *MGMT* regulatory elements (Figure 6). In MBM, methylation levels of promoter CpGs were strongly positively correlated, as were those of intragenic enhancer CpGs, whereas promoter and intragenic enhancer methylation levels were inversely correlated (Figure 6C). Methylation levels of intergenic enhancer CpGs also showed positive correlations, although less pronounced (Figure 6A). With the exception of a few CpGs in enhancer D (and one CpG in enhancer B), intergenic enhancer methylation was not associated with promoter methylation (Figure 6A).

Correlations between promoter and intragenic enhancer methylation levels were shared by MBM and GB (Figure 6C,D). In contrast, correlations involving intergenic enhancer methylation were more pronounced in MBM, with correlations for enhancer D detected nearly exclusively in MBM (Figure 6A,B).

### 3.5. Association of MGMT Promoter and Enhancer Methylation with MGMT Protein Expression

**Figure 7:**
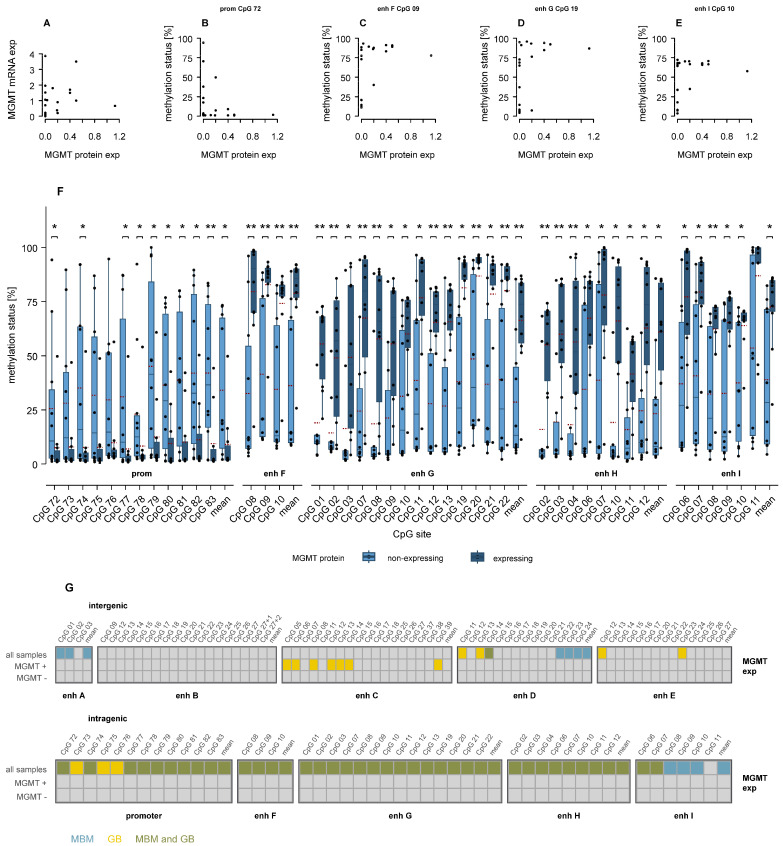
Divergent relationships of *MGMT* promoter and enhancer methylation with MGMT expression in MBM. (**A**) MGMT mRNA levels do not correlate linearly with protein expression. (**B**) Promoter methylation, e.g., at CpG 72, showed an L-shaped relationship with protein expression, (**C**–**E**) In contrast, CpGs in intergenic enhancers, e.g., CpG 09 in enhancer F (**C**), CpG 19 in enhancer G (**D**), and CpG 10 in enhancer I (**E**), showed an inverse L-shaped relationship with protein expression. (**F**) Boxplots indicating significant differences in promoter and intragenic enhancer methylation between MGMT expressing and non-expressing MBM samples. Mann–Whitney U test. Mean: red dashed line, * *p* ≤ 0.05, ** *p* < 0.01. (**G**) Summary of significant differences in methylation between MGMT expressing (MGMT +) and non-expressing (MGMT −) samples, with each box representing one CpG. Turquoise: MBM-specific, yellow: GB-specific, green: shared between MBM and GB. enh: enhancer.

For MBM samples, both MGMT mRNA and protein levels were available (Table 1, Figure 2). No linear correlation was observed between mRNA and protein expression (Figure 7A). In eight (40.0%) MBM samples, mRNA was detected despite the absence of MGMT protein.

None of the determined CpG methylation levels showed a linear correlation with MGMT mRNA or protein level. Instead, methylation of promoter CpGs displayed an L-shaped relationship with protein expression, exemplified by CpG 72 (Figure 7B), indicating that MGMT was only expressed in the absence of promoter methylation. In contrast, several intragenic enhancer CpGs exhibited an inverse L-shaped relationship with protein expression, as shown for CpGs in enhancers F, G, and I (Figure 7C–E), where low enhancer methylation was associated with loss of MGMT expression. Consistently, MBM samples expressing MGMT protein showed significantly higher intragenic enhancer methylation compared to non-expressing samples (*p* ≤ 0.05, Figure 7F), whereas intergenic enhancers displayed few significant differences (Figure S5).

Similar L-shaped and inverse L-shaped relationships, as well as increased enhancer methylation in promoter-methylated tumors, were observed in GB [26]. Tumor-specific and shared differences in promoter and intragenic enhancer methylation between MGMT-expressing and non-expressing samples are summarized in Figure 7G. Significant findings for MGMT protein-expressing samples only were unique to GB.

### 3.6. Association of MGMT Promoter and Enhancer Methylation with TERT Promoter Variants

**Figure 8:**
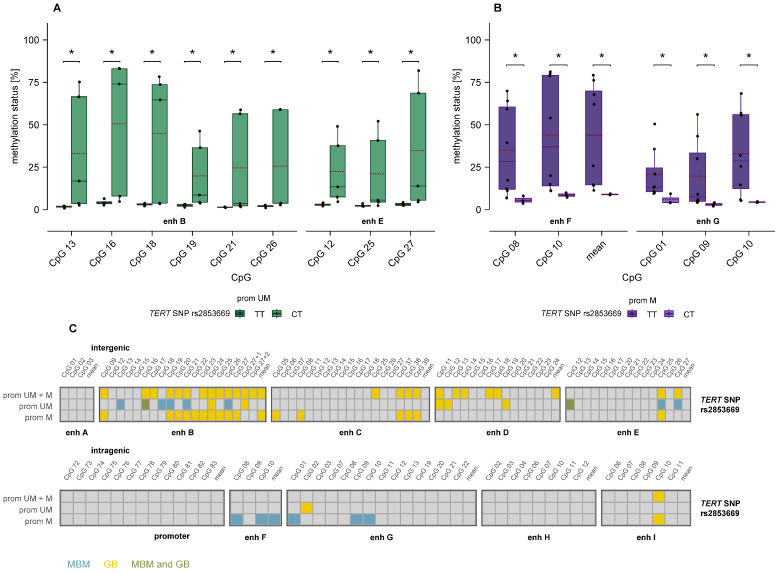
Association of *MGMT* promoter and enhancer methylation with *TERT* SNP rs2853669 in MBM. (**A**,**B**) Boxplots indicating significant differences in enhancer methylation between *TERT* SNP rs2853669 CT and TT genotypes, stratified by *MGMT* promoter methylation status: (**A**) unmethylated, (**B**) methylated. Mann–Whitney U test. Mean: red dashed line, * *p* ≤ 0.05. (**C**) Summary of associations in MBM and GB, with each box representing one CpG. Turquoise: MBM-specific, yellow: GB-specific, green: shared between MBM and GB. Abbreviations: prom UM: *MGMT* promoter-unmethylated, prom M: *MGMT* promoter-methylated, enh: enhancer.

We also examined associations between *MGMT* promoter and enhancer methylation and *TERT* promoter variants, including SNP rs2853669 and the promoter mutations C228T and C250T. For SNP rs2853669, TT and CT genotypes showed significant methylation differences in several CpGs in intergenic enhancers B and E in *MGMT* promoter-unmethylated samples (Figure 8A) and in intragenic enhancers F and G in promoter-methylated samples (Figure 8B). In contrast, no associations were observed between *MGMT* promoter or enhancer methylation and *TERT* promoter mutations C228T and C250T.

In GB, SNP rs2853669 genotypes TT and CT primarily differed in methylation of intergenic enhancers in promoter-methylated and unstratified samples [26], as summarized in Figure 8C. Consistent with MBM, no associations were detected between *MGMT* promoter or enhancer methylation and *TERT* promoter mutations C228T and C250T.

### 3.7. Association of MGMT Promoter and Enhancer Methylation with Overall Survival

**Figure 9:**
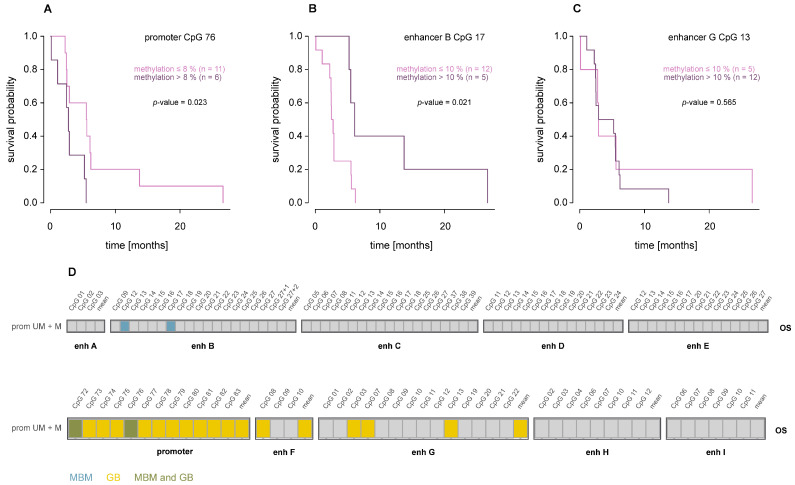
Association of *MGMT* promoter and enhancer methylation with overall survival in MBM. (**A**–**C**) Kaplan–Meier survival curves for MBM samples stratified by methylation status of promoter CpG 76 (**A**), enhancer B CpG 17 (**B**), and enhancer G CpG 13 (**C**). Patients were divided into two groups of roughly comparable size based on methylation levels. *p* values were determined by the log-rank test. (**D**) Summary of associations in MBM and GB, with each box representing one CpG. Turquoise: MBM-specific, yellow: GB-specific, green: shared between MBM and GB. Abbreviations: prom UM: *MGMT* promoter-unmethylated, prom M: *MGMT* promoter-methylated, enh: enhancer. Note that different cut-offs were used for MBM and GB.

In MBM, significant associations between CpG methylation and overall survival (OS) were observed only for the promoter (CpGs 72 and 76) and intergenic enhancer B (CpGs 12 and 17) (Figure 9A–D). However, these associations were only significant when patient MBM20 with an OS of 170.97 months was excluded as an outlier. Notably, these results hint at a survival benefit for MBM patients with lower promoter methylation (cut-off 8%) or higher enhancer B methylation (cut-off 10%). In GB, significant associations were detected for all 12 analyzed promoter CpGs as well as for several CpGs in intragenic enhancers F and G [26]. In contrast to MBM, higher promoter methylation and lower enhancer methylation were associated with improved survival.

### 3.8. Association of MGMT Promoter and Enhancer Methylation with Age and Sex

**Figure 10:**
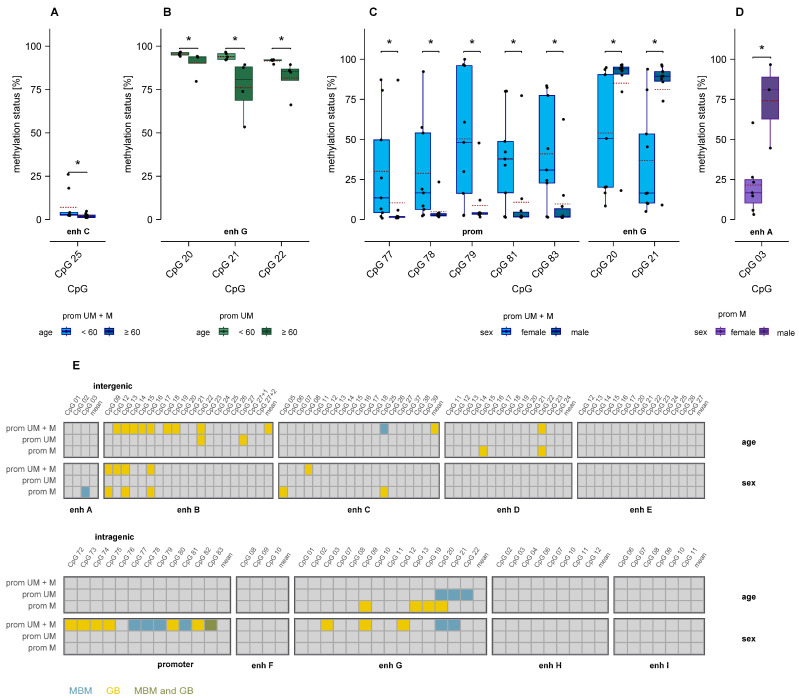
Association of *MGMT* promoter and enhancer methylation with age and sex in MBM. (**A**–**D**) Boxplots depicting significant differences in enhancer methylation between patients aged ≥ 60 years and < 60 years (**A**,**B**) and between female and male patients (**C**,**D**). Mann–Whitney U test. Mean: red dashed line, * *p* ≤ 0.05. (**E**) Summary of associations in MBM and GB, with each box representing an individual CpG. Colors indicate MBM-specific CpGs (turquoise), GB-specific CpGs (yellow), and CpGs shared between MBM and GB (green). Abbreviations: prom UM: *MGMT* promoter-unmethylated, prom M: *MGMT* promoter-methylated, enh: enhancer.

In MBM, samples from patients aged <60 years showed significantly higher methylation levels than those from elderly patients at one CpG in intergenic enhancer C in unstratified samples (Figure 10A) and at three CpGs in intragenic enhancer G in promoter-unmethylated samples (Figure 10B).

Females of the MBM cohort exhibited significantly higher methylation at five promoter CpGs and significantly lower methylation at two CpGs in intragenic enhancer G compared to males (*p* ≤ 0.05) in unstratified samples (Figure 10C). In promoter-methylated samples, males showed significantly higher methylation at a single CpG in enhancer A (*p* = 0.040, Figure 10D).

Significant age- and sex-specific associations of intergenic and intragenic enhancer methylation were also detected in GB, albeit at CpGs distinct from those observed in MBM [26] (Figure 10E).

## 4. Discussion

Growing evidence suggests that the brain microenvironment—composed of unique resident cell types, extracellular matrix components, and secreted factors—exerts a strong influence on the methylome of metastases [30]. Using immortalized cell cultures derived from 20 MBM and 34 GB patients, we aimed to gain insight into shared and tumor-specific methylation patterns in distinct brain malignancies. Importantly, no epigenetic drift was detected in the low-passage cell lines used in this study, confirming the reliability of the observed locus-specific methylation patterns. We focused on MBM and GB because both melanocytes and glial cells originate from neural crest cells [12]. Due to this common developmental origin and shared brain tissue environment, similar methylation features might be expected. However, the brain microenvironment is highly complex, and its composition varies markedly between different brain tumors [31]. Moreover, primary and secondary brain tumors differ in their evolutionary history and may therefore receive distinct microenvironmental cues [31]. Although *MGMT* methylation currently has limited clinical relevance in melanoma due to the advent of immunotherapy and targeted therapies, alkylating agents are still used in selected clinical settings. Moreover, understanding *MGMT* regulation provides insight into tumor biology.

We applied a targeted approach and determined methylation levels of 111 CpGs in *MGMT* regulatory elements and five CpGs of LINE-1, serving as a surrogate marker of global DNA methylation. We selected *MGMT* because promoter methylation is a well-established predictive marker of TMZ response in GB [32], while its relevance in MBM remains controversial [19,22]. In our MBM and GB cohorts, the proportion of promoter-methylated samples was almost identical (55.0% vs. 55.9%). Methylation levels of CpGs 72–83, the promoter CpGs regarded as functionally relevant in GB [33], were strongly correlated in both cohorts. Nevertheless, eight promoter CpGs exhibited significantly lower methylation in MBM compared to GB, whereas four CpGs showed no difference.

Our results suggest that *MGMT* promoter methylation contributes to gene regulation in both GB and MBM. In both tumor types, the relationship between promoter methylation and MGMT expression followed an L-shaped pattern: unmethylated promoters were associated with MGMT expression, whereas low to intermediate promoter methylation was linked to gene silencing. This relationship appeared less stringent in MBM, where 27.3% of promoter methylated samples still expressed MGMT and 22.2% of unmethylated samples lacked expression. By contrast, in GB, all promoter-methylated samples were MGMT-negative, and only 2.9% of tumors with unmethylated promoter lacked expression. Furthermore, in GB, MGMT non-expressing samples showed higher methylation in CpGs 72–83 than expressing samples, whereas in MBM, significant differences were restricted to nine CpGs. Together, these findings indicate that *MGMT* promoter methylation may exert a less important role in gene regulation in MBM than in GB.

We therefore examined whether enhancer methylation contributes to *MGMT* regulation in MBM. Aberrant enhancer methylation has been implicated in tumor progression and metastases [34,35], and enhancer methylation often predicts gene expression more accurately than promoter methylation [34]. Enhancers govern spatiotemporal gene expression patterns [36], but unlike promoters, their regulatory effects can extend over long genomic distances [37]. We therefore analyzed all *MGMT* enhancers listed in the VISTA Enhancer Browser [27], as well as an enhancer identified by Chen et al. [28]. In GB, we recently showed that methylation of these enhancers is associated with MGMT expression and with clinicopathological and demographic parameters [25,26,38].

Consistent with the promoter results, enhancer methylation was significantly lower in MBM than in GB, with the difference more pronounced for intergenic than intragenic enhancers. Specifically, 75% of intergenic CpGs and only 6.5% of intragenic CpGs exhibited lower methylation in MBM. In both tumor types, CpGs closer to the 5′ end of intergenic enhancers were more methylated than those located near the 3′ end, suggesting that CpG position contributes to distinct methylation patterns. Similar positional effects have been observed for CpGs in first introns, where unmethylated CpGs tend to occur near the 5′ end [39], although these regions are not classified as enhancers.

In MBM, CpGs within the *MGMT* promoter and enhancer D showed higher methylation levels compared to independent primary melanoma samples, whereas lower methylation levels were observed across all other enhancer regions. Our findings are in line with the literature reporting that due to tumor plasticity, methylation levels of MBMs differ from those of primary melanoma [35,40,41].

In both MBM and GB, methylation levels of intragenic enhancers were strongly positively correlated and inversely correlated with promoter methylation. Methylation levels of intergenic enhancer CpGs also correlated, though less strongly. Interestingly, correlations involving enhancer D were largely MBM-specific. Overall, methylation of intragenic enhancers appeared more homogenous and less tumor-type specific than that of intergenic enhancers.

In both brain malignancies, intragenic enhancer methylation was tightly linked to promoter methylation. At nearly all targeted CpGs in intragenic enhancers, MGMT-expressing samples showed higher methylation levels than non-expressing ones. Unlike promoter methylation, intragenic enhancer methylation showed an inverse L-shaped association with MGMT expression: low to intermediate methylation was linked to silencing, whereas high methylation was associated with MGMT expression. This finding indicates that enhancer methylation can influence gene expression in a context-dependent manner, rather than strictly following the classical model of DNA methylation–mediated repression.

Beyond epigenetic differences, structural genomic alterations may further contribute to the distinct regulatory patterns observed between GB and melanoma, including MBM. A major biological difference is the frequent loss of chromosome arm 10q in GB, where *MGMT* is located, resulting in *MGMT* hemizygosity. In this context, methylation of regulatory elements, including promoter and enhancer regions, may lead to more pronounced gene silencing. In MBM, where *MGMT* copy number loss is uncommon, residual expression may persist despite promoter or enhancer methylation, potentially explaining the weaker association between methylation and expression observed in MBM.

Growing evidence suggests that mutations in the *TERT* promoter occur early and frequently in cancer and play an important role in metastatic progression [42,43]. *TERT* encodes the rate-limiting catalytic subunit of telomerase, which maintains genomic integrity through telomere elongation [44]. In our MBM cohort, 45% exhibited a C250T mutation, 45% a C228T mutation, 5% a CC242TT mutation, and 5% carried the wildtype genotype. In GB, 32.4% and 58.8% of samples exhibited a C250T or C228T mutation, respectively, and 8.8% carried the wildtype genotype. Neither *MGMT* promoter nor enhancer methylation was associated with the occurrence of these mutations.

The SNP rs2853669 in the *TERT* promoter has also been linked to cancer risk and prognosis [45]. In our MBM cohort, 60% had the TT (wildtype) genotype and 40% the CT genotype, whereas in GB, 55.9% and 41.2% carried the respective genotypes (data from one sample were missing). In contrast to *MGMT* promoter methylation, enhancer methylation was associated with rs2853669 genotypes in both MBM and GB. In promoter-unmethylated MBM, the TT genotype was associated with lower methylation of several CpGs within intergenic enhancers B and E, whereas in promoter-methylated MBM, higher methylation was observed in CpGs within intragenic enhancers F and G. In GB, TT and CT genotypes primarily differed in the methylation of intergenic enhancers B, C, and D in promoter-methylated and unstratified samples. The association of enhancer rather than promoter methylation with rs2853669 genotypes may reflect a higher regulatory plasticity of enhancer regions in response to global chromatin alterations linked to telomerase activation.

In both GB and MBM, *MGMT* promoter and enhancer methylation were associated with OS. In GB, methylation of all 12 targeted promoter CpGs was linked to improved survival, consistent with the established predictive role of *MGMT* promoter methylation for TMZ response. In addition, lower methylation of one CpG and mean methylation of three CpGs in enhancer F and three individual CpGs, as well as mean methylation across 14 CpGs in enhancer G, were associated with improved OS. In our MBM cohort, associations were limited and less consistent than in GB. Notably, a single patient with an exceptionally long OS (171 months) strongly influenced the analysis. After exclusion of this outlier, lower methylation of promoter CpGs 72 and 76 and higher methylation of two CpGs in intergenic enhancer B were associated with improved OS. Our findings suggest that while *MGMT* methylation may have an impact on OS in MBM, its effect appears weaker and may be modulated by other tumor- or patient-specific factors. Due to the heterogeneity of treatments received, associations with OS should be interpreted with caution and confirmed in future studies.

Neither in MBM nor in GB was *MGMT* promoter methylation associated with patient age. In MBM, samples from patients aged < 60 years showed significantly higher methylation than those from patients aged ≥ 60 at one CpG in intergenic enhancer C (unstratified samples) and at three CpGs in intragenic enhancer G (promoter-unmethylated samples). In GB, age-related methylation differences occurred mainly in intergenic enhancer B and intragenic enhancer G. Age-associated methylation shifts, typically involving promoter hypermethylation and global hypomethylation, are common in tumors and reflect epigenetic drift with aging [46,47]. The limited number of affected CpGs in our study suggests that *MGMT* regulatory elements are relatively stable against such age-related changes in both tumor types.

Sex-specific differences were observed in methylation of several CpGs in the *MGMT* promoter, enhancer A–C, and enhancer G. Sex-related methylation differences have been described in various cancers and can influence gene regulation and therapy response. In GB, *MGMT* promoter methylation was found more frequently in females and had a stronger prognostic impact [48]. These findings suggest that sex-specific epigenetic regulation may contribute to the heterogeneity in methylation–expression relationships observed in brain malignancies.

Global hypomethylation has been implicated in tumor progression by promoting genome instability through demethylation of transposons and pericentromeric repeats and activation of oncogenes and has been associated with metastatic potential [49,50,51]. In this study, global DNA methylation was assessed by targeting five CpGs in LINE-1, a surrogate marker accounting for approximately 20% of the human genome. Global methylation was significantly lower in MBM than in GB, suggesting more pronounced epigenetic alterations in metastases. However, compared to independent primary melanoma samples, MBMs showed higher global methylation.

## 5. Conclusions

Our results reveal both shared and tumor-specific features of *MGMT* regulation in MBM and GB. Enhancer methylation was closely associated with promoter methylation, MGMT expression, and clinical as well as demographic parameters, highlighting the complexity of epigenetic regulation in brain malignancies. While some methylation–expression relationships are conserved across tumor types, others likely reflect tumor-specific biology or clinical behavior.

A key strength of our study is the use of identical methods for both MBM and GB cohorts within the same institutions, minimizing technical variability and ensuring that observed differences reflect true biological distinctions. The relatively low sample numbers remain a limitation, but our findings provide a robust framework for understanding *MGMT* epigenetic regulation across distinct brain tumors.

## Figures and Tables

**Table 1:** Characteristics of the MBM patient cohort.

Sample	Age [y]	Sex	MGMT Protein Expression	*MGMT* mRNA Expression	*TERT* Mutation	*TERT* SNP rs2853669	OS [m]	Therapy After Surgery
MBM01	39	male	0.00	1.96	wt	CT	6.08	radiation
MBM02	71	female	0.00	0.00	C228T	CT	2.76	none
MBM03 *	62	female	0.20	0.20	C250T	TT	26.66	radiation
MBM04	76	male	0.00	1.06	C250T	TT	1.08	none
MBM05	80	male	0.19	0.38	C228T	TT	2.5	radiation
MBM06	61	male	0.00	3.86	C228T	CT	2.24	chemotherapy with TMZ
MBM07	75	male	1.13	0.66	C228T	TT	5.52	n.a.
MBM08	49	male	0.03	1.03	CC242TT	CT	2.43	immunotherapy with Nivolumab
MBM09	62	male	0.40	1.50	C228T	TT	2.47	therapy with Roferon
MBM10	48	male	0.12	1.80	C250T	TT	6.21	chemotherapy with DTIC + Fotemustin
MBM11 *	70	female	0.00	0.15	C250T	TT	2.89	chemotherapy
MBM12 **	70	female	0.00	0.22	C250T	TT		
MBM13	38	female	0.20	0.90	C250T	TT	5.49	radiation, chemotherapy with DTIC
MBM14	59	female	0.00	1.52	C228T	CT	0.1	none
MBM15	40	male	0.40	1.70	C250T	TT	2.89	chemotherapy with Fotemustin + TMZ
MBM16	44	female	0.50	3.50	C228T	CT	13.74	radiation
MBM17	65	female	0.00	0.09	C250T	TT	5.23	n.a.
MBM18	41	male	0.00	0.00	C228T	CT	5.59	radiation
MBM19	61	female	0.50	1.00	C250T	CT	n.a.	n.a.
MBM20 *	50	female	0.00	0.70	C228T	TT	170.97	radiation

* first recurrence; ** second recurrence; wt: wildtype; y: years; OS: overall survival; n.a.: data not available. MBM11 and MBM12 originated from the same patient. Drugs: Temozolomide (TMZ), Nivolumab, Interferon alfa-2a (Roferon^®^), Dacarbazine (DTIC), Fotemustin.

## Data Availability

The original contributions presented in this study are included in the article/Supplementary Materials. Further inquiries can be directed to the corresponding author.

## Supplementary Materials

The following supporting information can be downloaded at: https://www.mdpi.com/article/10.3390/cells15050410/s1, Supplementary_information (Figures S1–S5, Tables S1–S6, R-packages).

## Abbreviations

The following abbreviations are used in this manuscript:

BM	brain metastases
CpG	CpG dinucleotide
enh	enhancer
GB	glioblastoma
LINE-1	Long Interspersed Nuclear Element-1
M	methylated
MBM	Melanoma brain metastases
MGMT	O^6^-methylguanine-DNA methyltransferase
OS	overall survival
PCR	polymerase chain reaction
prom	promoter
PSQ	pyrosequencing
SNP	single nucleotide polymorphism
TERT	telomerase reverse transcriptase
TMZ	temozolomide
UM	unmethylated
