# Supplementary material for: MGMT Promoter and Enhancer Methylation in Melanoma Brain Metastases and Glioblastoma: Shared and Distinct Features
Source: Cells. 2026 Feb 26;15(5):410. doi: 10.3390/cells15050410 (PMC12984658; doi:10.3390/cells15050410)
Supplement: Supplementary file 1 [file cells-15-00410-s001.zip › cells-4160934-supplementary.pdf]

Supplementary Information

MGMT Promoter and Enhancer Methylation in Melanoma Brain Metastases and Glioblastoma: Shared and Distinct Features

Katharina Pühringer [1,2], Benno Fehringer [1], Katja Zappe [1], Walter Berger [3], Serge Weis [4,5], Sabine Spiegl-Kreinecker [5,6] and Margit Cichna-Markl [1,*]

[1] Institute of Analytical Chemistry, Faculty of Chemistry, University of Vienna, 1090 Vienna, Austria;

[2] Vienna Doctoral School in Chemistry (DoSChem), University of Vienna, 1090 Vienna, Austria

[3] Center for Cancer Research and Comprehensive Cancer Center, Medical University of Vienna, 1090 Vienna, Austria

[4] Division of Neuropathology, Department of Pathology and Molecular Pathology, Kepler University Hospital GmbH, Johannes Kepler University, 4040 Linz, Austria

[5] Clinical Research Institute for Neurosciences, Johannes Kepler University, 4020 Linz, Austria

[6] Department of Neurosurgery, Kepler University Hospital GmbH, Johannes Kepler University, 4040 Linz, Austria

* Correspondence: margit.cichna@univie.ac.at

Supplementary Figures

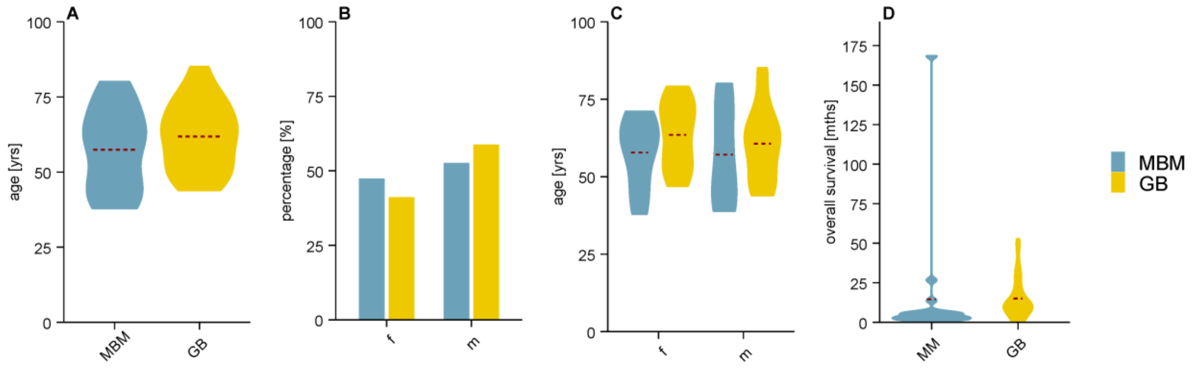

Figure S1. Distribution of age, sex and overall survival of the patients (f: female, m: male) in MBM and GB. Red dotted line: mean.

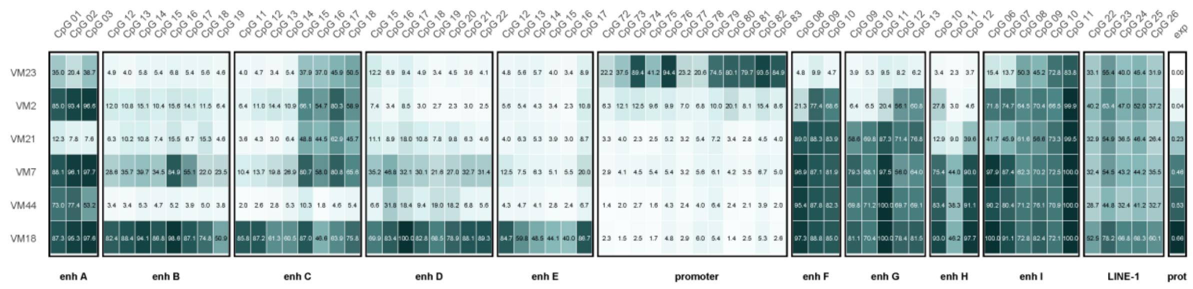

Figure S2. Heatmap of patient-specific methylation levels in melanoma. Methylation levels of the *MGMT* promoter, selected CpGs in intergenic enhancers A–E, intragenic enhancers F–I, and LINE-1 in six melanoma samples are shown. Samples are sorted by MGMT protein expression levels. Values represent means of two independent PCR/PSQ experiments. Color scale: white to dark green (0–100% methylation). enh: enhancer.

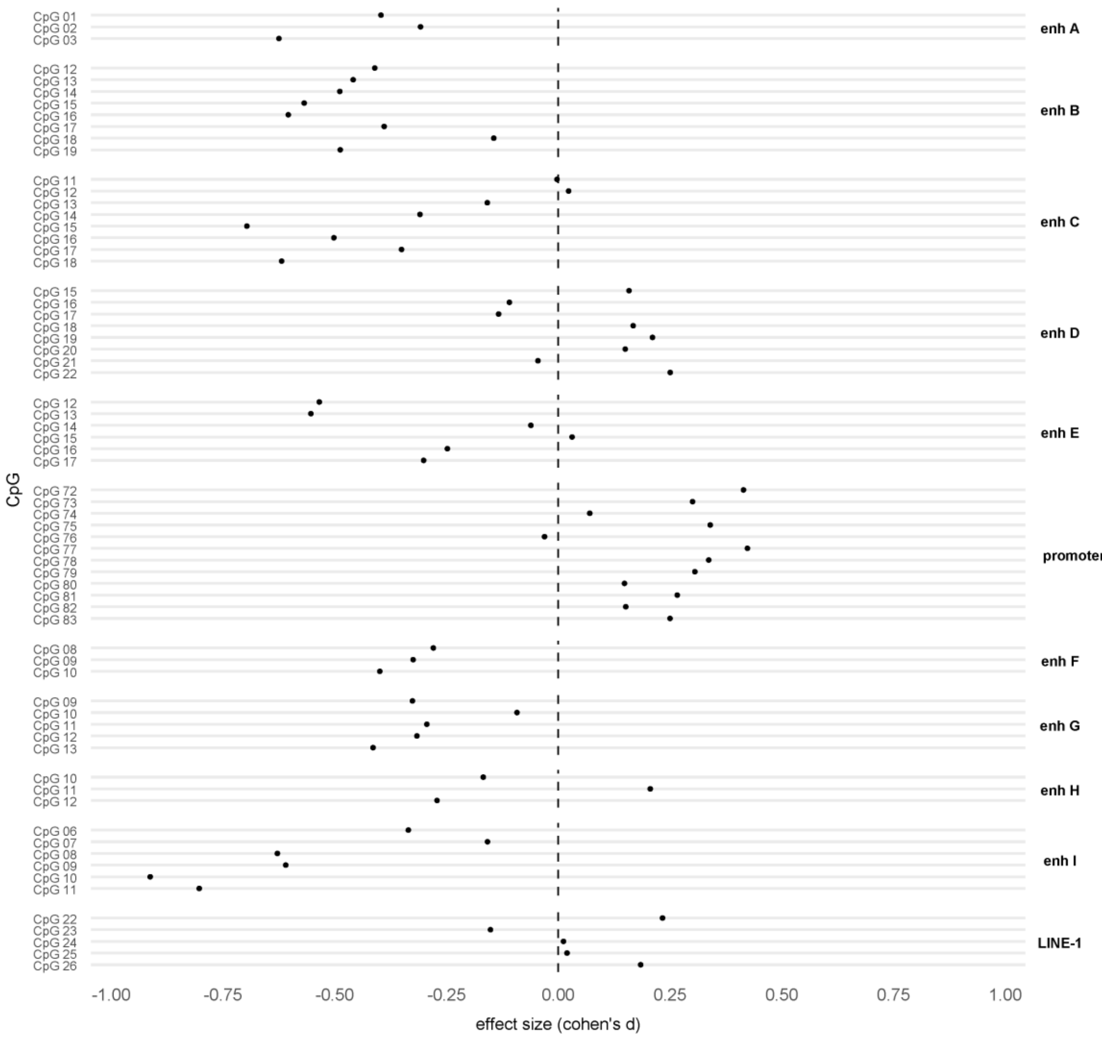

Figure S3. Forest plot, visualizing Cohen's d effect sizes (with Hedges' correction) for comparison of CpG methylation levels in MBM and melanoma samples in selected CpGs in intergenic enhancers A–E, intragenic enhancers F–I, the *MGMT* promoter, and LINE-1. Cohen's d < 0 describes lower and Cohen's d > 0 higher methylation levels in MBM compared to melanoma samples. enh: enhancer.

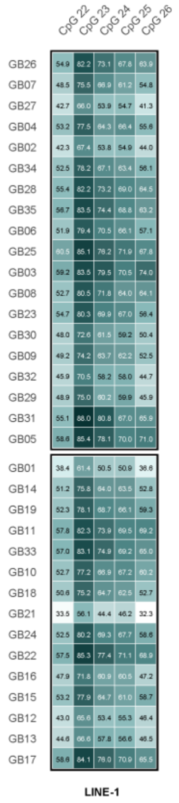

Figure S4. Methylation levels LINE-1 in GB. Samples are grouped by *MGMT* promoter methylation status (upper panel: methylated; lower panel: unmethylated) and further sorted by MGMT protein expression levels. Values represent means of two independent PCR/PSQ experiments. Color scale: white to dark green (0–100% methylation).

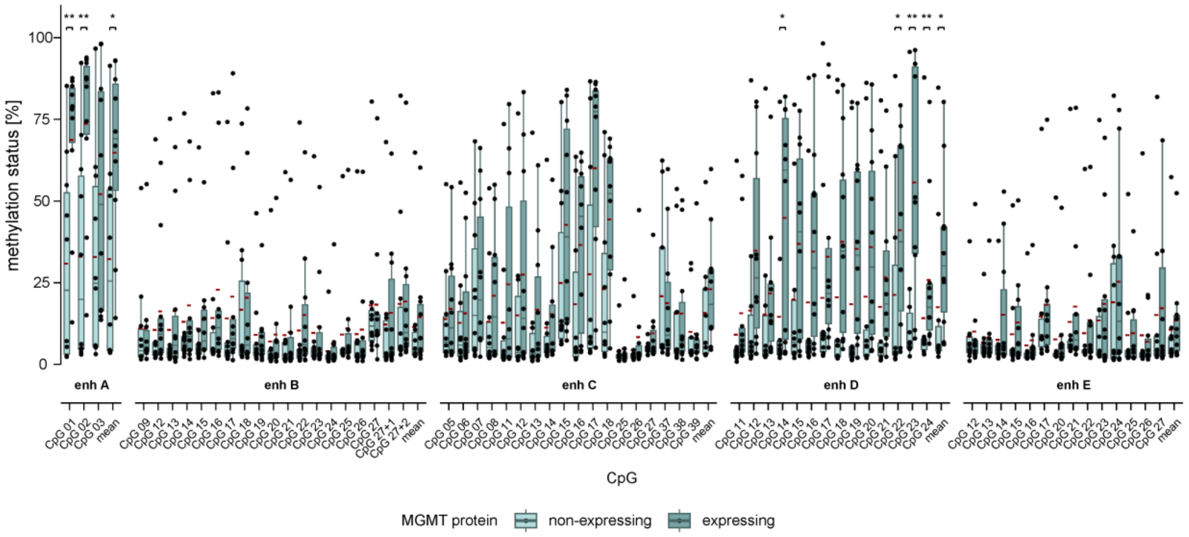

Figure S5. Distribution of methylation levels in intergenic enhancers in MBM. Samples stratified by MGMT protein expression levels (MGMT expressing/non-expressing in light/dark hues, respectively). Red dotted line: mean. Significance mark: *p≤0.05. enh: enhancer.

Supplementary Tables

Table S1 Summary of analyzed regulatory elements, respective coordinates (GRCh38.p14/hg38), coordinate source, location relative to the *MGMT* gene, element size, as well as the number of CpGs located in the respective elements.

**regulatory element**	**coordinates (GRCh38.p14/hg38)**	**location relative to *MGMT***	**source**	**size [bp]**	**CpGs**
enhancer A (hs542)	NC_000010.11:128165799-128166830	intergenic	VISTA Enhancer Browser	1032	8
enhancer B (hs737)	NC_000010.11:128568604–128569741	intergenic	VISTA Enhancer Browser	1138	27
enhancer C	NC_000010.11:128906630–128909942<br>Del 1: NC_000010.11:128906630–128908285<br>Del 2: NC_000010.11: 128908286–128909942	intergenic	[28]	3313	46
enhancer D (hs699)	NC_000010.11:129033193–129034911	intergenic	VISTA Enhancer Browser	1719	33
enhancer E (hs562)	NC_000010.11:129308258-129310478	intergenic	VISTA Enhancer Browser	2221	32
promoter	NC_000010.11:129466685–129467446	-	[25]	762	98
enhancer F (hs656)	NC_000010.11:129602684-129604015	intragenic	VISTA Enhancer Browser	1332	12
enhancer G (hs696)	NC_000010.11:129605804–129607046	intragenic	VISTA Enhancer Browser	1243	26
enhancer H (hs331)	NC_000010.11:129647523-129649421	intragenic	VISTA Enhancer Browser	1899	20
enhancer I (hs589)	NC_000010.11:129716557-129717922	intragenic	VISTA Enhancer Browser	1366	21
LINE-1	-	-	NCBI (GenBank: X58075.1)	1147	44

Table S2 Overview of PSQ assays applied in this study.

region	CpGs analyzed	Primer set	Primer sequence (5′→3′)	Amplicon length [bp]
**promoter**	72-83	A	F: GGATATGTTGGGATAGTT	98
98 CpGs			R: [Btn] CCCAAACACTCACCAAAT	
			S: GGATATGTTGGGATAGTT	
**enhancer A**	01-03	A	F: TGTTGGTTATTGTGAGTGATAGT	164
(hs542)			R: [Btn] AAACTCCATCCACTAAAACCTA	
8 CpGs			S: ATTTTTTGTTATGTAAAGT	
**enhancer B**	12-19	A	F: AGAATGTAGATTTGGGATTAGTTAAT	229
(hs737)			R: [Btn] TAAAATACAAAATATACCCTCCAACA	
27 CpGs			S: TATATAAAGAAGGTTGGT	
	09	C	F: [Btn] GTAGGTAGGGTTTGTGTAGTT	121
			R: TTAACTAATCCCAAATCTACATTCT	
			S: TCTACATTCTCTTCT	
	20, 21	D	F: AGGGATTTGGAGGGTAGGTGAA	97
			R: [Btn] TCAACTATCTACTTAACACAAACCAC	
			S: AGTTGTGTTTGTTTAGG	
	22-25	E	F: GTTGAGTGTGGTTTGTGTTAAGT	136
			R: [Btn] ACAACCTACCTCCTCTATACC	
			S: GTTAAGTAGATAGTTGA	
	26,27 +2	F	F: GGGTTTTGTGGAGGAATAATAAA	190
			R: [Btn] CTTTATACCTACAACATACTCCTA	
**enhancer C**	05–08	A	F: AGTTAGGAAATTAGAAATGGAATGTTT	255
(Chen *et al.*)			R: [Btn] CAAATCACACTCTAAATTCCCAATT	
46 CpGs			S: TGGTATTAGAGGTTA	
	11–18	B	F: [Btn] TTAAATAAGTGGTTTAGGTAGAGG	137
			R: TACTAAACATTCCATTTCTAATTTCC	
			S: CCATTTCTAATTTCCTAACTC	
	25–27	C	F: GTTGTAGGGTATATGAGTTTAGAT	271
			R: [Btn] TTCATAACTCAAATTAACACACACT	
			S: TTTTGTGTTGAATGG	
	37–39	D	F: GAGGTTATTTGGAAAGTTGAGAT	286
			R: [Btn] CTAATAATCCAAACCCTCTATTC	
			S: TTTAGTGTTATGGGAG	
**enhancer D**	11, 12	B	F: AATTAAATTGTTAAGTAGGTATTAGAG	141
(hs699)			R: [Btn] AAATATCAAACTCTCAAATCATCCT	
33 CpGs			S: TAGGTATTAGAGGTTG	
	13, 14	C	F: TTGGTTTGTGTGTTATTTATAGTAT	169
			R: [Btn] TCCTCAATATTCAAACTATCATAATA	
			S: GTTGAATTTAGTTTTTGTAA	

region	CpGs analyzed	Primer set	Primer sequence (5′→3′)	Amplicon length [bp]
	15–22	A	F: TGTGTTAGTTTTAGTGGTTTAGA<br>R: [Btn] TAACACACAAACCAATCTCTC<br>S: TAGTTTTAGTGGTTTAGAAGT	138
	23, 24	D	F: AGTTATTGGAGTTGGTATAATAGA<br>R: [Btn] ATCTCTTATTCTAATTCAACCTTCA<br>S: ATTATGGAAAGATTAAT	167
**enhancer E**<br>(hs562)<br>32 CpGs	12-17	A	F: TGTGGTAGTGGTATTTTTTAGTTAGTAGA<br>R: [Btn] CCATTTTAATATACAACACTTTCCCTTTTT<br>S: AATTATTTTTTATGATTGTTA	268
	20-22	B	F: TTTGTATATTGAAGAGGGAAGAAGAA<br>R: [Btn] AAAAAAAATAATTACTCTAACAACCCTTT<br>S: ATTATTTTTTATATTTTAG	134
	23-27	C	F: ATTGTAAGGGAAAGGGTTGTTAGAGTAA<br>R: [Btn] AACTCCCCTACAAACTTTTCACCCTAC<br>S: AGGTTTTGTTTATTGTAATTTTATG	221
**enhancer F**<br>(hs656)<br>12 CpGs	08-10	A	F: TTAATGATTTTTAGTTGTTTGTTGT<br>R: [Btn] TACTATTATTATACATATCCAAATAAA<br>S: AGATGATTAGTAAGTGAGA	198
**enhancer G**<br>(hs696)<br>26 CpGs	01–03	A	F: [Btn] GGAATGTGTTATTTAATTGGTATGT<br>R: CAAATCCCACAACAAATCCTTAT<br>S: TCAAAAAAAAAAAATCACC	204
	07–08	B	F: GAGGTTTGATATAAGTAATGATGG<br>R: [Btn] CCTCCTAATCCCACAATACAA<br>S: TAAGTAATGATGGTATG	131
	09–13	C	F: AGGTTTGATATAAGTAATGATGGTAT<br>R: [Btn] CRTATTCTCTCCCACTTCAATA<br>S: GTATTGTGGGATTAGGA	257
	19–22	D	F: [Btn] GTGTATTGAAGTGGGAGAGAATA<br>R: CAATAACAATTTTACAAACACAAATAACTT<br>S: ATAACTTTTCATTCA	241
**enhancer H**<br>(hs331)<br>20 CpGs	02-04	A	F: GGGTTGTTAGAAAGTTGATGAAA<br>R: [Btn] AAAAAAAACAATTCATCTCCCTTTAT<br>S: AGAAAGTTGATGAAATG	180
	06, 07	B	F: ATTGAATTATGGGTTTATTTAAAATGGT<br>R: [Btn] ACACTTTCCACCTTTTACTATCA<br>S: GAGGTTAAAATTTTTATAGATTGGA	249
	10-12	C	F: GATTTTTTAAATTAGTGAAGAGTGGATATT<br>R: [Btn] AAATCCCACAACCACAAAAAAAAACAA	182

region	CpGs analyzed	Primer set	Primer sequence (5′→3′)	Amplicon length [bp]
			S: TTAAATTAGTGAAGAGTGGATATT	
**enhancer I**	06-11	A	F: TGGTTTAAGTTATTTAGGTTGAATGTTAAT	209
(hs589)			R: [Btn] CTACTCATTAAAAATACCATCACTCAAAT	
21 CpGs			S: ATTGAGTATATTTTATTAAGAA	
**LINE 1**	22-26		F: AGGTGTGGGATATAGTYT	73
44 CpGs			R: CACTCCCACCCRAATAT	
			S: AGGTGTGGGATATAGTYT	

[Btn]: biotin; length: PCR product length; bp: base pairs; F: forward primer; R: reverse primer; S: sequencing primer; Y: C/T, R: G/A.

Table S3 Clinical and demographic data of the GB patient cohort.

patient	age [y]	sex	MGMT protein expression	*TERT* mutation	*TERT* rs2853669	OS [m]	primary therapy	adjuvant therapy
GB01	64	female	0.00	C250T	TT	7.43	R-Ch-T	none
GB02	85	male	0.00	wt	TT	3.00	none	none
GB03	53	female	0.00	C228T	CT	52.50	R-Ch-T	none
GB04	67	female	0.00	C228T	TT	46.63	RT	none
GB05	57	female	0.00	C228T	TT	10.50	RT	CCNU
GB06	46	male	0.00	C228T	TT	30.50	R-Ch-T	TMZ + CCNU
GB07	50	female	0.00	C250T	n.s.	27.40	RT	TMZ
GB08	74	female	0.00	C228T	CT	7.79	R-Ch-T[1]	Avastin + TMZ
GB09	48	male	0.00	wt	TT	1.55	none	none
GB10	64	male	0.20	C228T	CT	11.70	R-Ch-T	TMZ
GB11	73	female	0.16	C228T	TT	10.60	R-Ch-T	no
GB12	44	male	1.10	C250T	CT	13.00	R-Ch-T	TMZ
GB13	65	male	1.20	C228T	TT	9.27	RT[1]	none
GB14	69	male	0.04	C250T	TT	8.00	RT	TMZ + CCNU
GB15	73	female	1.10	C250T	CT	7.00	none	none
GB16	83	male	1.00	C228T	CT	9.57	RT	none
GB17	74	male	1.32	C250T	CT	5.19	R-Ch-T	none
GB18	44	male	0.25	wt	TT	23.15	R-Ch-T	TMZ
GB19	48	male	0.09	C228T	CT	18.67	R-Ch-T	TMZ
GB21	60	male	0.40	C228T	TT	13.00	R-Ch-T	Avastin
GB22	53	male	0.54	C250T	TT	0.89	none	none
GB23	47	female	0.00	C250T	CT	37.25	R-Ch-T	TMZ
GB24	64	male	0.49	C228T	CT	7.73	R-Ch-T	TMZ
GB25	67	female	0.00	C228T	TT	16.80	R-Ch-T	TMZ
GB26	75	female	0.00	C228T	CT	8.22	RT	none

patient	age [y]	sex	MGMT protein expression	*TERT* mutation	*TERT* rs2853669	OS [m]	primary therapy	adjuvant therapy
GB27	52	female	0.00	C250T	TT	n.a.	R-Ch-T	TMZ
GB28	63	male	0.00	C250T	CT	21.80	R-Ch-T	TMZ
GB29	79	female	0.00	C228T	CT	1.31	none	none
GB30	58	male	0.00	C228T	TT	16.00	R-Ch-T	TMZ
GB31	58	female	0.00	C228T	TT	13.30	R-Ch-T	TMZ
GB32	71	male	0.00	C228T	TT	1.25	none	none
GB33	57	male	0.20	C250T	CT	12.70	R-Ch-T	TMZ
GB34	53	male	0.00	C228T	TT	32.30	R-Ch-T	TMZ
GB35	64	male	0.00	C228T	TT	10.60	R-Ch-T	none

wt: wildtype; y: years; OS: overall survival; n.a.: data not available, Drugs: Lomustine (CCNU), temozolomide (TMZ), Bevacizumab (Avastin), Ch-T: chemotherapy, R-Ch-T: radio-chemotherapy, RT: radiotherapy.
[1] therapy had to be discontinued

Table S4 Characteristics of the melanoma patient cohort.

sample	age [y]	sex	MGMT protein expression	OS [m]
VM7	74	female	0.46	6.08
VM21	46	male	0.23	2.76
VM44	46	female	0.53	26.66
VM2	28	male	0.04	1.08
VM18	65	female	0.66	2.5
VM23	47	male	0.00	2.24

Table S5 Cohen's d effect sizes (with Hedges' correction) for comparing selected CpG methylation levels in the MBM and melanoma cohorts in intergenic enhancers. Cohen's d < 0 describes lower and Cohen's d > 0 higher methylation levels in MBM compared to melanoma samples.

regulatory element	CpG	effect size (Cohen's d)
enhancer A	01	-0.40
	02	-0.31
	03	-0.62
enhancer B	12	-0.41
	13	-0.46
	14	-0.49
	15	-0.57
	16	-0.60
	17	-0.39
	18	-0.14
	19	-0.49
enhancer C	11	0.00
	12	0.02
	13	-0.16
	14	-0.31
	15	-0.70
	16	-0.50
	17	-0.35
	18	-0.62
enhancer D	15	0.16
	16	-0.11
	17	-0.13
	18	0.17
	19	0.21
	20	0.15
	21	-0.05
	22	0.25
enhancer E	12	-0.53
	13	-0.55
	14	-0.06
	15	0.03
	16	-0.25
	17	-0.30

Table S6 Cohen's d effect sizes (with Hedges' correction) for comparing CpG methylation levels in the MBM and melanoma cohorts in the *MGMT* promoter and intragenic enhancers. Cohen's d < 0 describes lower and Cohen's d > 0 higher methylation levels in MBM compared to melanoma samples.

regulatory element	CpG	effect size (Cohen's d)
promoter	72	0.41
	73	0.30
	74	0.07
	75	0.34
	76	-0.03
	77	0.42
	78	0.34
	79	0.31
	80	0.15
	81	0.27
	82	0.15
	83	0.25
enhancer F	08	-0.28
	09	-0.32
	10	-0.40
enhancer G	09	-0.33
	10	-0.09
	11	-0.29
	12	-0.32
	13	-0.41
enhancer H	10	-0.17
	11	0.21
	12	-0.27
enhancer I	06	-0.34
	07	-0.16
	08	-0.63
	09	-0.61
	10	-0.91
	11	-0.80
LINE-1	22	0.23
	23	-0.15
	24	0.01
	25	0.02
	26	0.18

Supplementary R-packages

Wickham et al. (2019). Welcome to the tidyverse. Journal of Open Source Software, 4(43), 1686. https://doi.org/10.21105/joss.01686.

Taiyun Wei and Viliam Simko (2021). R package 'corrplot': Visualization of a Correlation Matrix (Version 0.92). https://CRAN.R-project.org/package=corrplot

Hadley Wickham (2016). ggplot2: Elegant Graphics for Data Analysis. Springer-Verlag New York. https://ggplot2.tidyverse.org

Alboukadel Kassambra (2023). ggpubr: 'ggplot2' Based Publication Ready Plots. R-package version 0.6.0. https://CRAN.R-project.org/package=ggpubr

William Revelle (2024). psych: Procedures for Psychological, Psychometric, and Personality Research. Northwestern University, Evanston, Illinois. R package version 2.4.6. https://CRAN.R-project.org/package=ggsignif

Baptiste Auguie. (2017). gridExtra: Miscellaneous functions for "grid" graphics (Version 2.3). https://CRAN.R-project.org/package=gridExtra

David Robinson, Alex Hayes and Simon Couch (2022). broom: Convert Statistical Objects into Tidy Tibbles. R package version 1.0.6. https://CRAN.R-project.org/package=broom

Winston Chang W (2023). extrafont: Tools for Using Fonts. R package version 0.19. https://CRAN.R-project.org/package=extrafont

Torchiano M (2020). effsize: Efficient Effect Size Computation. R package version 0.8.1. https://CRAN.R-project.org/package=effsize
